# *Legionella pneumophila* Strain 130b Evades Macrophage Cell Death Independent of the Effector SidF in the Absence of Flagellin

**DOI:** 10.3389/fcimb.2017.00035

**Published:** 2017-02-16

**Authors:** Mary Speir, Adam Vogrin, Azadeh Seidi, Gilu Abraham, Stéphane Hunot, Qingqing Han, Gerald W. Dorn, Seth L. Masters, Richard A. Flavell, James E. Vince, Thomas Naderer

**Affiliations:** ^1^Department of Biochemistry and Molecular Biology, Biomedicine Discovery Institute, Monash UniversityClayton, VIC, Australia; ^2^Department of Immunobiology, Howard Hughes Medical Institute, Yale University School of MedicineNew Haven, CT, USA; ^3^Centre National de la Recherche Scientifique, Institut National de la Santé et de la Recherche Médicale, Institut du Cerveau et la Moelle - Hôpital Pitié-Salpêtrière, Boulevard de l'hôpital, Sorbonne Universités, UPMC Univ Paris 06Paris, France; ^4^Department of Medicine, Center for Pharmacogenomics, Washington University School of MedicineSt. Louis, MO, USA; ^5^Walter and Eliza Hall Institute of Medical ResearchParkville, VIC, Australia; ^6^Department of Medical Biology, University of MelbourneParkville, VIC, Australia

**Keywords:** infection, bacteria, pyroptosis, caspases, pneumonia, mitochondria, live-cell imaging

## Abstract

The human pathogen *Legionella pneumophila* must evade host cell death signaling to enable replication in lung macrophages and to cause disease. After bacterial growth, however, *L. pneumophila* is thought to induce apoptosis during egress from macrophages. The bacterial effector protein, SidF, has been shown to control host cell survival and death by inhibiting pro-apoptotic BNIP3 and BCL-RAMBO signaling. Using live-cell imaging to follow the *L. pneumophila*-macrophage interaction, we now demonstrate that *L. pneumophila* evades host cell apoptosis independent of SidF. In the absence of SidF, *L. pneumophila* was able to replicate, cause loss of mitochondria membrane potential, kill macrophages, and establish infections in lungs of mice. Consistent with this, deletion of BNIP3 and BCL-RAMBO did not affect intracellular *L. pneumophila* replication, macrophage death rates, and *in vivo* bacterial virulence. Abrogating mitochondrial cell death by genetic deletion of the effectors of intrinsic apoptosis, BAX, and BAK, or the regulator of mitochondrial permeability transition pore formation, cyclophilin-D, did not affect bacterial growth or the initial killing of macrophages. Loss of BAX and BAK only marginally limited the ability of *L. pneumophila* to efficiently kill all macrophages over extended periods. *L. pneumophila* induced killing of macrophages was delayed in the absence of capsase-11 mediated pyroptosis. Together, our data demonstrate that *L. pneumophila* evades host cell death responses independently of SidF during replication and can induce pyroptosis to kill macrophages in a timely manner.

## Introduction

*Legionella pneumophila* is the aetiological agent of Legionnaires' Disease, a potentially life-threatening form of pneumonia in the elderly and immuno-compromised individuals (Cunha et al., 2016). Infection is initiated by inhaling aerosols derived from *L. pneumophila* contaminated water sources, such as cooling towers. Within the lungs, *L. pneumophila* establishes a specialized niche, termed the *Legionella*-containing vacuole, in resident alveolar macrophages, which promotes immune protection and bacterial growth. Virulence is absolutely dependent on the Type IV secretion system (T4SS). Many of the over 300 effector proteins translocated by the T4SS hijack host cell processes, including apoptotic cell death pathways, important for intracellular survival (Isberg et al., 2009; Speir et al., 2014).

Apoptosis is a tightly regulated program of cellular suicide depending on the activation of cytosolic cysteine-dependent aspartic acid-specific proteases, such as caspase-3. In the case of intrinsic apoptosis, the pro- and anti-apoptotic members of the BCL-2 protein family control the activity of the sentinel cell death regulators, BAX, and BAK (Czabotar et al., 2014). Activation of BAX and/or BAK leads to the loss of mitochondrial membrane integrity and release of cytochrome-*c*, which nucleates apoptosome formation to activate caspase-9 (Youle and Strasser, 2008). Active caspase-9 then cleaves caspases-3 and -7, which initiate dismantling of the cell via proteolysis of essential proteins (Elmore, 2007). Cellular stresses, including bacterial infections, can promote activation of the pro-apoptotic BH3-only proteins that either directly, or indirectly, induce BAX/BAK-mediated apoptosis (Chipuk et al., 2010). This effectively controls intracellular pathogens by compromising their replicative niche and triggering bacterial clearance in a cell autonomous manner (Chow et al., 2016).

*L. pneumophila* primarily replicates in macrophages and, thus, depends critically upon the health of its host cell for survival. For example, detection of flagellin results in the rapid induction of caspase-1 dependent pyroptotic cell death, which prevents *L. pneumophila* replication and infection in mice (Molofsky et al., 2006; Ren et al., 2006; Zamboni et al., 2006; Miao et al., 2010; Zhao et al., 2011). Similarly, *L. pneumophila* must also prevent mitochondrial apoptosis to promote replication, as loss of pro-survival BCL-2 family members, BCL-XL and MCL-1, induces cell death of infected macrophages and *Legionella* clearance in lungs (Speir et al., 2016). How *Legionella* evades apoptosis remains unresolved as *L. pneumophila* can trigger caspase-3 activation during macrophage invasion without inducing immediate cell death (Molmeret et al., 2004; Abu-Zant et al., 2005). *L. pneumophila* induces the transcriptional up-regulation of several pro-survival BCL-2 family members in a T4SS dependent manner, but, paradoxically, also pro-apoptotic factors, such as BNIP3 (Losick and Isberg, 2006b; Abu-Zant et al., 2007). While BNIP3 activity can be blocked by BCL-2, it can target mitochondria directly and induce cell death independently of BCL-2 (Zhang and Ney, 2009). SidF is the only *L. pneumophila* effector identified to bind and inhibit the activity of BNIP3, as well as BCL-RAMBO, which may similarly induce death in a BCL-2-dependent and -independent manner (Kataoka et al., 2001; Banga et al., 2007). Consistent with this, loss off SidF was reported to result in increased apoptotic cell death of *L. pneumophila* infected macrophages, raising the possibility that BNIP3 and BCL-RAMBO are important host factors that control *L. pneumophila* (Banga et al., 2007). In late stages of *L. pneumophila* infections, macrophages are characterized by nucleic acid fragmentation and activated apoptotic caspases, suggesting that *L. pneumophila* induces BAX/BAK-dependent apoptosis during escape (Abu-Zant et al., 2005; Santic et al., 2005; Fischer et al., 2006). Also, *L. pneumophila* infected lungs of susceptible mice show apoptosis associated phenotypes (Santic et al., 2007). While at least five effectors have been identified that can activate mitochondria-mediated apoptosis in macrophages, combined deletion of these effectors did not abrogate growth in macrophage or resulted in reduced caspase-3 activity (Nogueira et al., 2009; Zhu et al., 2013).

Besides apoptosis and flagellin/caspase-1 mediated pyroptosis, cytosolic contamination with *L. pneumophila* lipopolysaccharide triggers the activation of caspase-11, independent of flagellin, which subsequently cleaves Gasdermin D to form pores in the plasma membrane (Case et al., 2013; Casson et al., 2013; Shi et al., 2015). Caspase-11 can also activate the NLRP3/caspase-1 inflammasome and caspase-1 the apoptotic caspase-7 to control cell death-independent mechanisms as observed in *L. pneumophila* infection (Akhter et al., 2009; Case et al., 2013; Casson et al., 2013; Cerqueira et al., 2015), suggesting that during *L. pneumophila* infections caspase activation can affect multiple cellular events.

To gain a better understanding of the role of apoptosis in *L. pneumophila* infection, we have established a novel imaging method that allows for single-cell analysis of *L. pneumophila*-infected macrophages in real-time. This enables the identification of subtle and transient host-pathogen interactions, which may be overlooked in traditional methods that extrapolate from only a small number of isolated data points, or only analyze cells at the population level. With this technique, we now show that *L. pneumophila* is able to replicate and induce normal macrophage killing rates in the absence of SidF, as well as BNIP3 and BCL-RAMBO. Consistent with this, loss of SidF, BNIP3, or BCL-RAMBO did not affect *L. pneumophila* lung infections in mice. Moreover, we show that mitochondrial apoptosis itself is not essential for the induction of host macrophage cell death, nor for bacterial replication.

## Materials and methods

### Ethics statement

Animal experiments were performed in accordance with the National Health and Medical Research Council Australian Code of Practice for the Care and Use of Animals and were approved by the Monash University Animal Ethics Committee (approval number 2011/086), and by the Walter and Eliza Hall Institute Animal Ethics Committee. All mice were maintained under specific pathogen-free conditions. Age- and sex-matched mice were chosen to be included in different treatment groups without randomization.

### *Legionella pneumophila* strains

*Legionella pneumophila* 130b serogroup 1 (ATCC BAA-74) is a spectinomycin-resistant clinical isolate from the Wadsworth Veterans Administration Hospital, Los Angeles, CA (Edelstein, 1986). The avirulent Δ*dotA* and the flagellin-deficient Δ*flaA* strains are deletion mutants of *L. pneumophila* 130 b. To generate the Δ*flaA*/Δ*sidF* deletion strain, ~500 bp fragments from upstream and downstream of sidF (LPW28321) were amplified and fused together using overlap extension PCR. The construct was cloned into the Sal1 site of the plasmid pSR47S, and Δ*flaA L. pneumophila* were transformed to select for kanamycin resistant clones. The second integration to delete the entire *sidF* coding region was selected for on 10% sucrose plates and individual colonies were verified by PCR for loss of SidF. The complemented Δ*flaA*/Δ*sidF* strains were generated by cloning the full-length sidF gene into the plasmid pMMB207C. All *L. pneumophila* strains were grown from −80°C frozen stocks on buffered charcoal-yeast extract (BCYE) agar at 37°C for 48 h before each infection. To determine bacterial numbers, *L. pneumophila* were re-suspended in PBS to determine optical density at 600 nm (OD_600_), whereby an OD_600_ of 1 equaled 10^9^ bacteria/mL. Based on this, macrophages were infected with multiplicity of infections (MOI) of 10, unless otherwise indicated.

### Cell culture

Murine bone marrow-derived macrophages (BMDMs) were obtained from femora and tibiae of female 6–8 week-old C57BL/6 mice, or from mice of the indicated genotypes. Macrophages were cultured in RPMI 1640 medium supplemented with 15% fetal bovine serum (Serana), 20% L-cell-conditioned medium (containing macrophage colony-stimulating factor), and 100 U/mL of penicillin-streptomycin (Sigma) in bacteriological dishes for 7 days, at 37°C + 5% CO_2_. For infections, BMDMs were gently scraped from plates using a cell scraper (BD Falcon) and washed three times in PBS, before seeding into tissue culture-treated plates.

### Live-cell imaging to determine macrophage viability

To follow *Legionella* infection in real-time using live-cell imaging, macrophages (2.5 × 10^5^ cells/mL) were seeded into 96-well tissue culture-treated plates. Before infection, BMDMs were stained with 1 μM Cell Tracker Green (CTG) (Invitrogen) for 20 min in serum-free RPMI 1640. Medium was then replaced with RPMI 1640 supplemented with 15% FBS and 10% L-cell-conditioned medium containing 50 nM tetramethylrhodamine (TMRM) and 600 nM Draq7 (Abcam). Cells were infected with *L. pneumophila* strains at a MOI of 10. In some experiments, the CellEvent Casaspase-3/7 detection reagent (Invitrogen) was added to measure caspase activity by time-lapse imaging. Before imaging, 50 μL of mineral oil (Sigma) was added to each well to prevent evaporation.

Experiments were performed on a Leica AF6000 LX epi-fluorescence microscope equipped with an incubator chamber set at 37°C + 5% CO_2_ and an inverted, fully-motorized stage driven by Leica Advanced Suite Application software. Time-lapse images were acquired with bright-field, GFP, TxRed, and Y5 filters every hour for up to 72 h using a 10 × /0.8-A objective. To determine the percentage of dead cells, images were analyzed in ImageJ and in MetaMorph (Molecular Devices) using a custom-made journal suite incorporating the count nuclei function to segment and count the number of CTG, TMRM, caspase active and Draq7-positive cells (adapted from Croker et al., 2011). The data was analyzed in Excel and GraphPad Prism.

### Measurement of colony-forming units (CFUs)

To determine bacterial burdens, macrophages were seeded at a density of 2.5 × 10^5^ cells/mL into 12-well tissue culture plates and infected with *L. pneumophila* strains at an MOI of 5. After 2 h, cells were washed 3 × in PBS and the medium replaced. For analysis, cells were lysed in 0.05% digitonin for 5 min at room temperature and serial dilutions of the cell lysates and the corresponding culture media were plated on BCYE agar plates. Bacterial colonies were counted after 72 h at 37°C.

### Mice infections

C57BL/6 mice were obtained from Monash Animal Research Platform (MARP). BNIP3^−/−^ (Diwan et al., 2007), BAK^−/−^, BAX/BAK^−/−^ (Willis et al., 2007), Ppif^−/−^ (Baines et al., 2005), Casp1/11^−/−^ and Casp-11^−/−^ (Kayagaki et al., 2013) mice have been characterized previously. A targeting vector for BCL-RAMBO was generated from a 129/Sv genomic library, linearized and transfected into TC-1 embryonic stem (ES) cells by electroporation. Southern blotting was used to identify ES cell clones with homologous recombination, which were used for injection into blastocysts to generate chimeric mice. Chimeric mice were bred at least 10 generations onto the C57BL/6 background and were deficient in BCL-RAMBO expression (SI Figure 1).

Six to eight week-old male or female mice, in groups of five or more, were anesthetized by 4% isofluorane inhalation and infected intra-nasally with 2.5 × 10^6^ *L. pneumophila* in 50 μL of sterile PBS. For CFUs, at 6 or 48 h following infection, both lung lobes were removed and homogenized for 30 s in PBS at 30,000 rpm using the Omni Tissue Master homogenizer. Serial dilutions of the lung homogenates were plated onto BCYE agar plates and bacterial colonies were counted after 72 h at 37°C to determine CFUs.

### Immunoblot analysis

2.5 × 10^5^ cells were lysed in 120 μL SDS-loading dye, boiled for 5 min, and samples analyzed by 12% SDS-PAGE. After transfer to nitrocellulose membranes (Millipore), membranes were blocked with 5% skim milk in T-BST (Tween-20, Tris-buffer) for 1 h at room temperature. Membranes were probed with anti-cleaved caspase-3 antibody (CST #9964) or anti-β-actin antibody (Millipore #04-1116) (loading control) and re-suspended in T-BST + 5% skim milk, overnight at 4 °C. After washing, membranes were probed with secondary goat anti-rabbit IgG (Life Technologies) and goat anti-mouse IgG (Life Technologies) antibodies conjugated to HRP (1:20,000 dilution in T-BST + 5% skim milk). Membranes were developed with the luminol-based enhanced chemiluminescence (ECL) and exposed to film (Kodak). Scanned images were processed in Photoshop Adobe.

### Statistical analyses

For all *in vitro* data, two-way analysis of variance was performed before using Tukey's *post hoc* test for pairwise comparisons. For mice infections, data were analyzed by the Mann-Whitney *U*-test. In all experiments, *p* ≤ 0.05 were taken to be significant.

## Results

### Live-cell imaging of *L. pneumophila* infected macrophages

**Figure 1:**
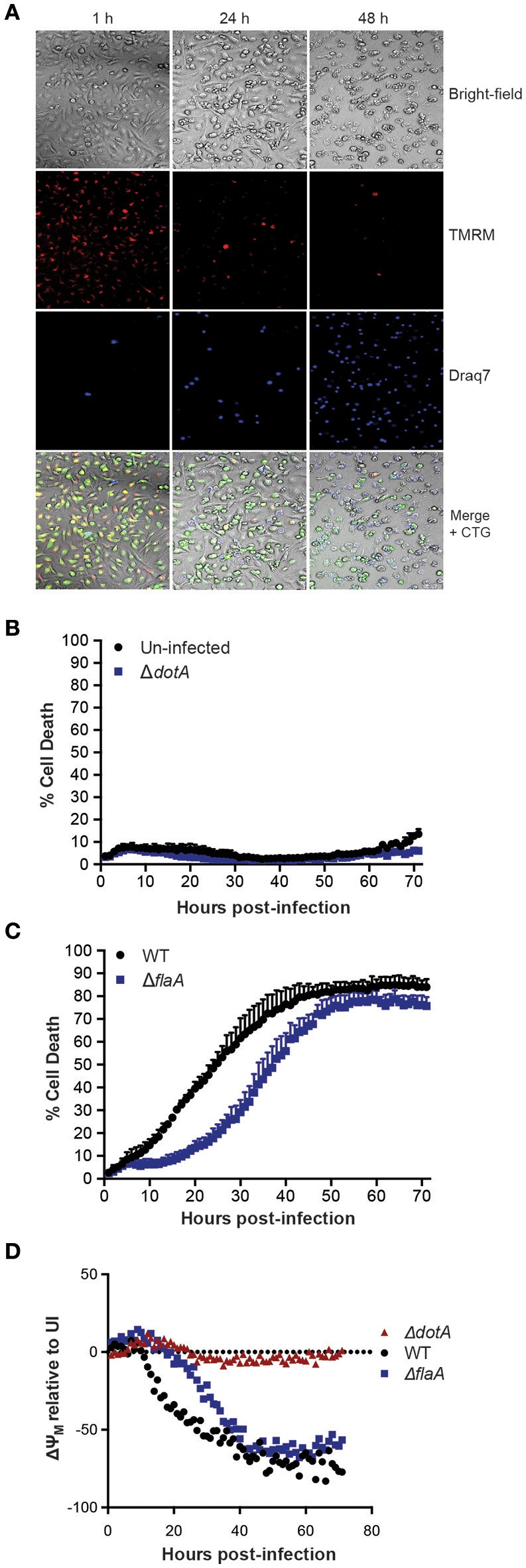
**Live cell imaging of macrophage and mitochondrial health in *L. pneumophila* infections. (A)** Wild type C57BL/6 bone marrow-derived macrophages (BMDMs) labeled with the fluorescent dyes tetramethylrhodamine methyl ester (TMRM) to stain active mitochondria and Draq7 to detect dead cells were infected at an MOI of 10 with Δ*flaA L. pneumophila*. Live-cell images from 1, 24, and 48 h post-infection are shown. **(B)** Draq7 positive (dead) uninfected and Δ*dotA L. pneumophila* infected BMDMs determined by live-cell imaging over 72 h. **(C)** Draq7 positive (dead) WT and Δ*flaA L. pneumophila* infected BMDMs. **(D)** TMRM fluorescence (ΔY_M_, mitochondrial membrane potential) of WT, Δ*dotA*, and Δ*flaA L. pneumophila* treated BMDMs over 72 h. TMRM fluorescence intensity is relative to that in uninfected BMDMs (dotted line). Mean and SD. of three independent biological replicates are shown.

To examine *L. pneumophila* infection of bone marrow-derived macrophages (BMDMs) in real-time and to monitor their viability, infected cells were stained with the cell-permeable fluorescent dye, tetramethylrhodamine methyl ester (TMRM), which is sequestered by active mitochondria, depending on the inner membrane potential. In addition, the macrophage culture media contained the membrane impermeable DNA fluorophore Draq7 to specifically stain dead cells (Figure 1A). Importantly, more than 90% of BMDMs left uninfected, or infected with the avirulent Δ*dotA* strain, which lacks a functional T4SS, did not show uptake of Draq7 and remained viable for up to 72 h, demonstrating that it is possible to follow host-pathogen interactions over extended time periods (Figure 1B). As expected, the BMDMs infected with WT *L. pneumophila* died more rapidly than those infected with the flagellin-deficient strain, Δ*flaA*, consistent with a flagellin/caspase-1-mediated pyroptotic cell death. For example, at 30 h post infection more than 60% of WT-infected BMDMs were Draq7-positive, whereas <30% of the Δ*flaA* infected BMDMs (Figure 1C). Over time, 80% of BMDMs infected with WT or Δ*flaA L. pneumophila* were killed by 72 h post infection, consistent with repeated rounds of bacterial infection, egress, and re-infection. However, only Δ*flaA L. pneumophila* is able to replicate in BMDMs, demonstrating that *L. pneumophila* critically depends on evading macrophage death during early stages of infections for growth. In addition to Draq7 staining, we monitored mitochondrial membrane potential (ΔΨm) over time. The Δ*dot*-infected BMDMs showed little change in TMRM fluorescence, similar to that of uninfected BMDMs (Figure 1D). In contrast, in WT- and Δ*flaA*-infected BMDMs the ΔΨm decreased by more that 50% relative to uninfected BMDMs (Figure 1D). Comparable to the Draq7 uptake, loss of ΔΨm occurred more quickly in the BMDMs infected with WT *L. pneumophila* than in those infected with Δ*flaA L. pneumophila* (Figure 1D). These results demonstrate that live-cell fluorescent imaging, to follow Draq7- and TMRM-staining to quantify cell death and mitochondrial integrity, respectively, is able to distinguish between the different cell death kinetics involved in *L. pneumophila* infection.

### Loss of the bacterial effector SidF does not lead to increased apoptosis of infected macrophages

**Figure 2:**
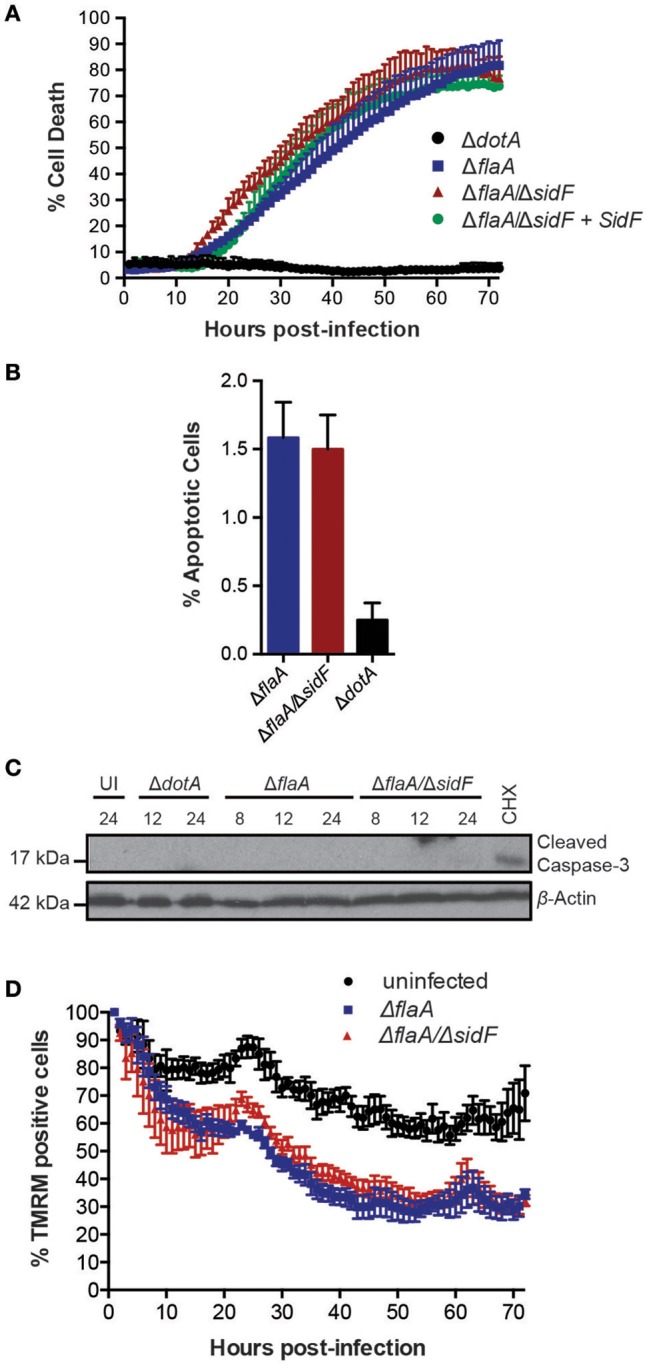
**Loss of SidF does not induce apoptotic cell death of infected BMDMs. (A)** Draq7 positive (dead) BMDMs infected at a MOI of 10 with Δ*dotA*, Δ*flaA*, Δ*flaA/*Δ*sidF*, and Δ*flaA/*Δ*sidF* + SidF *L. pneumophila*. Data are representative of three independent experiments. Mean and S.D. of three independent biological replicates shown. **(B)** Percentage apoptotic cells as determined by membrane blebbing in BMDMs infected with Δ*dotA*, Δ*flaA*, and Δ*flaA*/Δ*sidF L. pneumophila*. >800 cells were scored from live-cell images taken every 30 min for 48 h. Mean and S.D. shown. **(C)** Time course immuno-blot analysis for cleaved (indicative of active) caspase-3 in BMDMs infected with Δ*flaA* or Δ*flaA/*Δ*sidF L. pneumophila*. BMDMs treated with 10 μM cycloheximide (CHX) were used as a positive control. Actin blot is a loading control. **(D)** TMRM fluorescence of uninfected BMDMs or infected with Δ*flaA* and Δ*flaA/*Δ*sidF L. pneumophila* over 72 h. Mean and SEM from two independent experiments containing three biological repeats shown.

**Figure 3:**
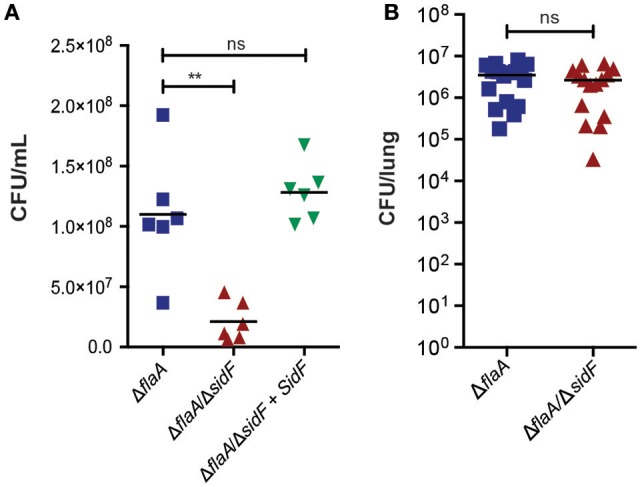
***L. pneumophila*** **is able to infect lungs in the absence of SidF. (A)** BMDMs were infected with the Δ*flaA*, Δ*flaA/*Δ*sidF*, and Δ*flaA/*Δ*sidF* + SidF *L. pneumophila* and bacterial burdens (CFU/mL) calculated 48 post infection. Symbols represent biological repeats from two independent experiments. Line indicates mean. ^**^p < 0.01, ns, not significant **(B)** C57BL/6 mice were infected intranasal with 2.5 × 10^6^ Δ*flaA* and Δ*flaA*/Δ*sidF L. pneumophila* and lung bacterial burdens (CFU/lung) determined 48 h post infection. Symbols represent data from individual mice from two independent experiments. Line indicates mean. ns = not significant.

The *L. pneumophila* effector protein SidF is the only effector reported to target and inhibit host cell pro-apoptotic factors (Banga et al., 2007). To investigate its role in *Legionella* induced killing of BMDMs and to avoid rapid pyroptotic cell death, we generated a Δ*flaA*/Δ*sidF Legionella* mutant. Surprisingly, we did not observe any significant increase in the rate or extent of cell death in BMDMs infected with the Δ*flaA*/Δ*sidF* strain compared to Δ*flaA* or the complemented Δ*flaA*/Δ*sidF* strain (Figure 2A). Although loss of SidF did result in increased BMDM death at 20 h post infection, this was not significantly different to the Δ*flaA*- and complemented Δ*flaA*/Δ*sidF*-induced killing (Figure 2A). Furthermore, <2% of the Δ*flaA*/Δ*sidF*-infected BMDMs underwent apoptotic cell death during the first 24 h of infection, as judged by cell shrinkage and membrane blebbing, which was similar to the number of Δ*flaA*-infected BMDMs (Figure 2B). Consistent with this finding, there was no detectable caspase-3 cleavage, indicative of apoptotic caspase activation, in either the Δ*flaA*- or Δ*flaA*/Δ*sidF*-infected BMDMs after 8, 12, and 24 h of infection, as determined by immunoblotting of the caspase-3 p17/p19 fragment (Figure 2C). Finally, infection with the Δ*flaA*/Δ*sidF L. pneumophila* strain did not result in increased mitochondrial damage compared to Δ*flaA* over 72 h (Figure 3D). Taken together, these data demonstrate that loss of SidF does not result in a dramatic induction of apoptosis or increased BMDM death in *L. pneumophila* infections.

### SidF is dispensable for *L. pneumophila* replication in macrophages

Given our observations that SidF does not play a major role in preventing death of BMDMs, we next tested whether it is required for replication of *L. pneumophila*, as previously reported (Banga et al., 2007). We determined bacterial burdens (CFU/mL) from BMDMs infected with Δ*flaA*, Δ*flaA*/Δ*sidF*, or Δ*flaA*/Δ*sidF* + SidF *L. pneumophila* at 48 h post-infection (Figure 3A). After 48 h of infection, there was a small but significant difference (≃3-fold; *p* < 0.01) between the CFU/mL recovered from the Δ*flaA*/Δ*sidF* strain compared to the Δ*flaA* or Δ*flaA*/Δ*sidF* + SidF *L. pneumophila* strains in BMDMs, which was less obvious in immortalized BMDMs that support rapid bacterial growth (SI Figure 2). Given this observed, albeit marginal, growth defect in BMDMs, the role of SidF was further examined in *L. pneumophila* infections *in vivo*. Bacterial burdens from the lungs of WT C57BL/6 mice 48 h after infection with either Δ*flaA* or Δ*flaA*/Δ*sidF L. pneumophila* were not significantly different (Figure 3B). This demonstrates that, while SidF promotes bacterial replication to a small degree *in vitro*, it is dispensable for *L. pneumophila* survival and burdens during lung infections in mice.

### BCL-RAMBO and BNIP3 deficiency do not affect *L. pneumophila* induced macrophage death

**Figure 4:**
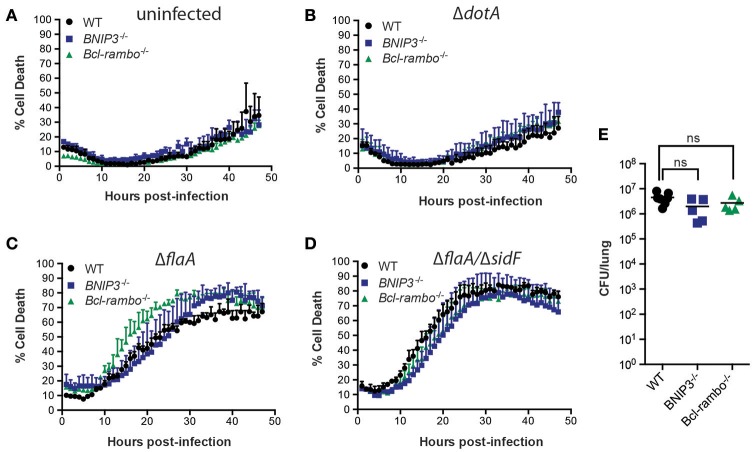
***L. pneumophila*** **infects macrophages and mice independent of BNIP3 and BCL-RAMBO**. Draq7 positive (dead) uninfected **(A)**, Δ*dotA* **(B)**, Δ*flaA* **(C)**, Δ*flaA/*Δ*sidF* **(D)** infected C57BL/6 wild-type (WT), BCL-RAMBO^−/−^, and BNIP3^−/−^ BMDMs. Data are representative of three independent experiments. Mean and S.D. of three independent biological replicates shown. **(E)** Bacterial lung burdens (CFU/lung) of C57BL/6 wild-type (WT), BNIP3^−/−^, and BCL-RAMBO^−/−^ mice infected intranasal with 2.5 × 10^6^ Δ*flaA L. pneumophila* 48 h post infection. Data from individual mice and mean (line) are shown. Ns, not significant.

SidF has been reported to target and inhibit BCL-RAMBO and BNIP3 (Banga et al., 2007), which may act independently to modulate cell death in both a caspase-dependent and -independent manner (Kim et al., 2011; Rikka et al., 2011). To test whether BCL-RAMBO and BNIP3 play role in *L. pneumophila* infection, we utilized *BCL-RAMBO*^−/−^ and *BNIP3*^−/−^ mice. As expected, loss of BNIP3 or BCL-RAMBO did not affect the viability of uninfected or Δ*dotA*-*L. pneumophila* infected BMDMs over 72 h (Figures 4A,B). Loss of BNIP3 did not influence Δ*flaA L. pneumophila*-induced killing of BMDMs (Figure 4C), which was marginally, but not significantly, increased in BCL-RAMBO deficient BMDMs (Figure 4C). Finally, loss of BNIP3 or BCL-RAMBO did not affect Δ*flaA*/Δ*sidF*-induced killing of BMDMs, but reduced the increased death rates observed in Δ*flaA*-infected BCL-RAMBO deficient BMDMs (Figure 4D). This demonstrates that even in the absence of SidF, BNIP3, and BCL-RAMBO are dispensable for *L. pneumophila*-mediated killing of macrophages. To confirm that BCL-RAMBO or BNIP3 do not contribute to *L. pneumophila* infection *in vivo*, bacterial burdens were calculated from the lungs of WT C57BL/6, *BCL-RAMBO*^−/−^, and *BNIP3*^−/−^ mice 48 h after infection with Δ*flaA L. pneumophila*. As shown in Figure 4E, there was no significant difference in bacterial numbers recovered from the lungs of the different mouse genotypes (*p* > 0.05). In agreement with this, Δ*flaA* and Δ*flaA*/Δ*sidF* replication in *BCL-RAMBO*^−/−^ macrophages was similar compared to WT macrophages (SI Figure 2).

### *L. pneumophila* induces macrophage cell death independently of Cyclophilin-D and BAX/BAK

**Figure 5:**
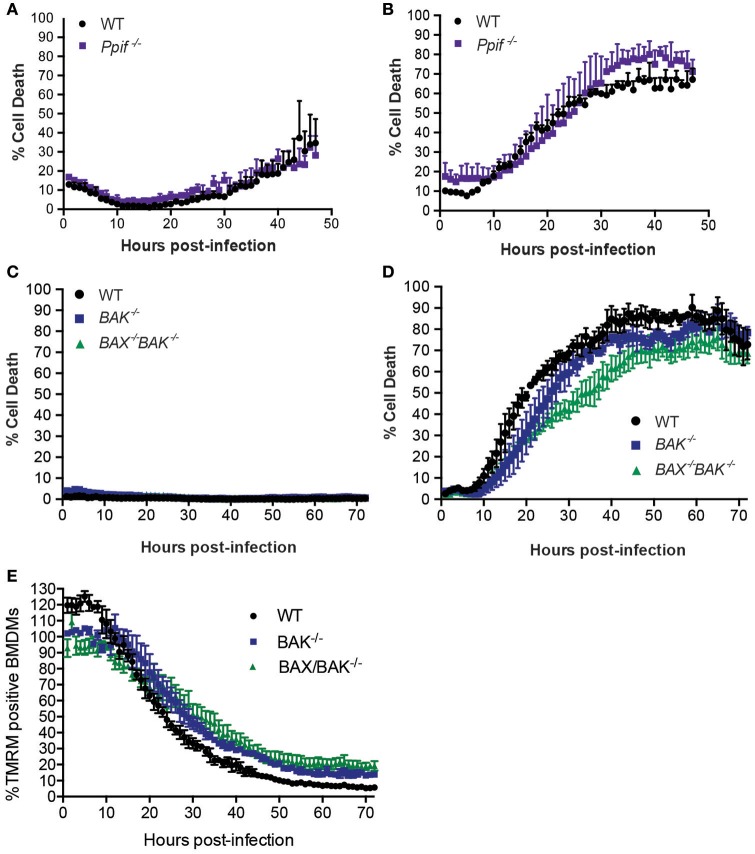
***L. pneumophila*** **induces macrophage death independent of Cyclophilin-D, BAX, and BAK**. Draq7 positive (dead) Δ*dotA* **(A)** and Δ*flaA L. pneumophila* **(B)** infected wild-type (WT) and *Ppif* ^−/−^ BMDMs. Draq7 positive (dead) Δ*dotA* **(C)** and Δ*flaA L. pneumophila* **(D)** infected (MOI of 10) wild-type (WT), *BAK*^−/−^ and *BAX*^−/−^ *BAK*^−/−^ BMDMs. Mean and S.D. of three biological replicates shown. **(E)** TMRM positive BMDMs infected with Δ*flaA L. pneumophila* (relative to Δ*dotA* infected BMDMs) over 72 h. Mean and SD from three biological repeats, representative of at least two independent experiments, shown.

Although BNIP3 and BCL-RAMBO do not influence *Legionella* infection, alternate regulators of mitochondria-mediated cell death signaling may be targeted by *Legionella* to promote bacterial replication or egress. These include host cell death mediated by formation of the mitochondrial permeability transition pore (mPT) (Khemiri et al., 2008), or intrinsic (mitochondrial) apoptosis, mediated by BAX and BAK oligomerisation on mitochondrial membranes. The *Ppif* gene product, Cyclophilin-D (CycD), is a critical component of the mPT, can modulate apoptosis independent of BCL-2 and may be targeted by BNIP3 (Carneiro et al., 2009; Gutiérrez-Aguilar and Baines, 2015). We therefore tested the role of CycD in *L. pneumophila* infection. As expected, Δ*dotA* infected CycD-deficient BMDMs (*Ppif* ^−/−^) remained viable for 72 h (Figure 5A). In the absence of CycD, Δ*flaA*-infected BMDMs remained viable for the first 10 h post infection and then showed increased cell death that were indistinguishable from infected WT BMDMs (Figure 5B). This result demonstrates that *L. pneumophila* can still induce cell death normally in the absence of a functional mitochondrial permeability transition pore complex.

To test whether *Legionella* infection is influenced by a loss of intrinsic (mitochondrial) apoptosis, we utilized BMDMs deficient in BAK alone, or both BAX and BAK, which has been demonstrated to completely prevent intrinsic apoptotic cell death (van Delft et al., 2006). As expected, in the absence of BAK, or BAX and BAK together, Δ*dotA L. pneumophila*-infected BMDMs remained viable (Figure 5C), indicating that neither BAX nor BAK were essential to cell survival under these conditions. Infection with Δ*flaA L. pneumophila* resulted in the death of similar numbers of *BAK*^−/−^ and *BAX*^−/−^*BAK*^−/−^ BMDMs by 72 h post infection (Figure 5D). Deletion of both BAX and BAK resulted in a 15–20% decrease in the rate of cell death compared to WT, or deletion of BAK alone, between 30 and 40 h post infection, but not during the initial killing phase (10–25 h). In addition, the rate loss of TMRM signal was similar between WT, *BAK*^−/−^, and *BAX*^−/−^*BAK*^−/−^ BMDMs, suggesting that BAX and BAK do not significantly contribute to loss of mitochondrial membrane potential in *L. pneumophila* infections (Figure 5E). Consistent with this, we and other have recently shown that Δ*flaA L. pneumophila* replicates normally in *BAX*^−/−^*BAK*^−/−^ BMDMs (Nogueira et al., 2009; Speir et al., 2016). Together, these data suggest that while Δ*flaA L. pneumophila* can induce BAX/BAK-mediated apoptosis in late stage infections, it is not critical for bacterial replication.

### Inhibition of host protein synthesis by *L. pneumophila* does not lead to apoptosis, but pyroptosis

**Figure 6:**
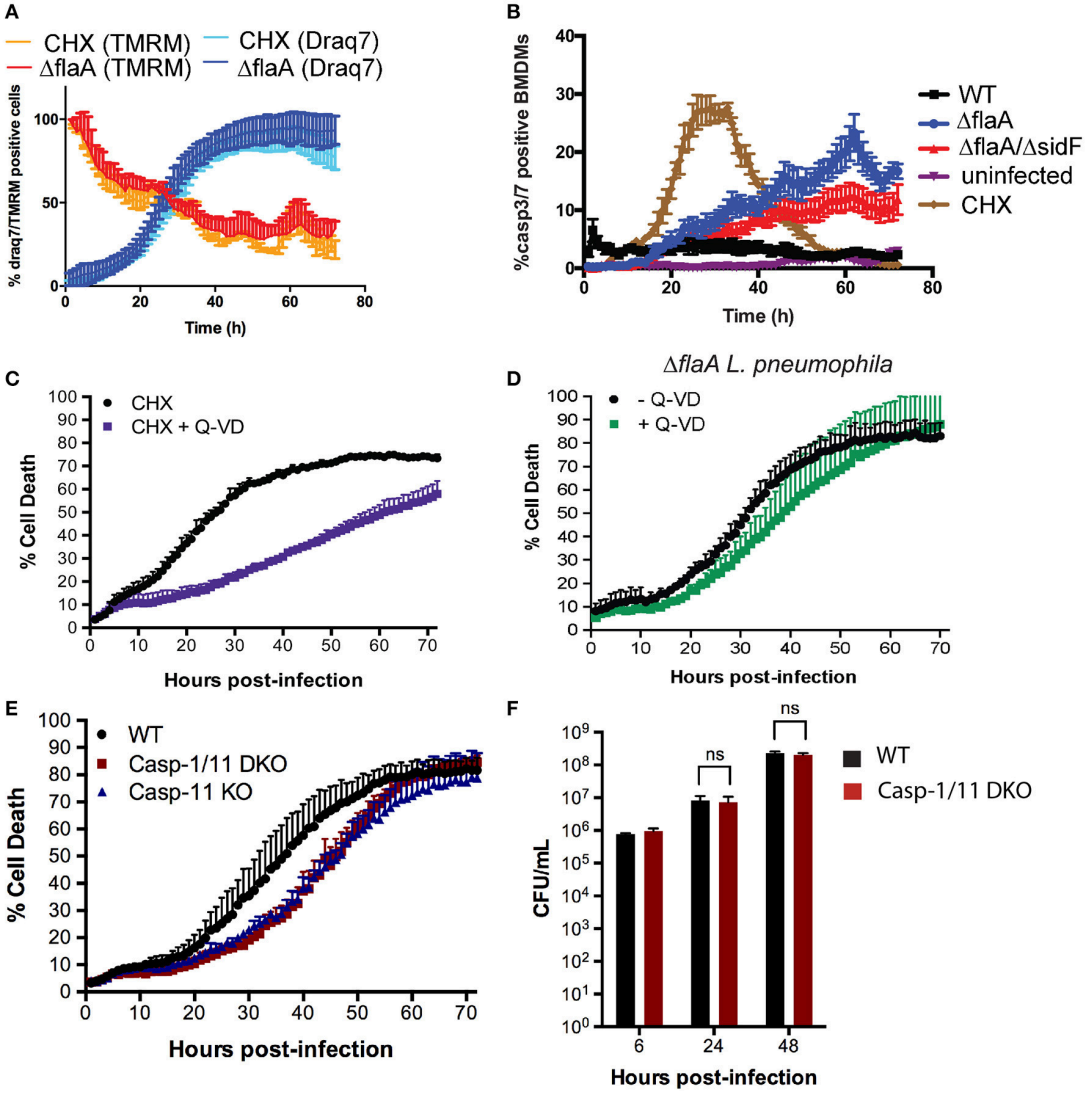
***L. pneumophila*** **mediated macrophage death is independent of apoptotic caspases, but induced by pyroptotic caspase-11. (A)** Draq7 and TMRM positive BMDMs treated with cycloheximide (2 μg/ml) or infected with Δ*flaA L. pneumophila*. **(B)** Caspase-3/7 activity in uninfected, WT, Δ*flaA* and Δ*flaA/*Δ*sdiF L. pneumophila* infected or cycloheximide (CHX, 2 μg/ml) treated BMDMs. **(C)** Draq7 positive (dead) BMDMs treated with cycloheximide (CHX) with or without Q-VD (20 μM). **(D)** Draq7 positive (dead) BMDMs infected with Δ*flaA L. pneumophila* and treatment with or without Q-VD (20 μM). Data are representative of three independent experiments. Mean and S.D. of three independent biological replicates shown. **(E)** Draq7 positive (dead) WT, Caspase-1/11 DKO and Caspase-11 KO BMDMs infected with Δ*flaA L. pneumophila*. Data are representative of two independent experiments. Mean and S.D. of three independent biological replicates shown. **(F)** Bacterial burdens (CFU/mL) from Δ*flaA L. pneumophila*-infected WT and Caspase-1/11 DKO BMDMs at 6, 24, and 48 h post-infection. Mean and S.E.M of three independent experiments shown.

We have recently shown that *L. pneumophila* limits host cell protein synthesis to reduce levels of the short-lived pro-survival BCL-2 family member MCL-1, akin to chemically inhibiting protein synthesis by cycloheximide (CHX) treatment (Speir et al., 2016). Δ*flaA L. pneumophila* infection or CHX treatment also reduced the mitochondrial membrane potential and induced cell death with similar kinetics at the MOI and concentrations used (Figure 6A). To follow the activation of apoptotic caspases on a single cell level over extended periods, BMDMs were incubated with a fluorescent probe to detect caspase-3/7 activity. As expected, CHX treatment caused activation of caspase-3/7 at around 20 h post treatment, coinciding with cell death (Figure 6B, SI Video 1). In contrast, Δ*flaA L. pneumophila* induced cell death with minimal caspase-3/7 activity (Figure 6B, SI Video 2, consistent with western blot analysis (Figure 2C). Only at late stage infections did Δ*flaA L. pneumophila* cause caspase3/7 activation (Figure 6B), at which point most BMDMs were stained by Draq7 and thus contained compromised membranes (Figure 6A). Similar results were obtained with Δ*flaA/sidF L. pneumophila*, whereas uninfected BMDMs remained viable with little evidence of caspase-3/7 activity (Figure 6B). WT *L. pneumophila* caused sustained low levels of caspase-3/7 activity immediately after infections (Figure 6B, SI Video 3).

CHX induces mitochondrial apoptosis, which is delayed by the pan-caspase inhibitor, QVD-ph (QVD) for at least for 30 h (Figure 6C). In contrast, QVD treatment had only a marginal effect on Δ*flaA L. pneumophila* induced killing of BMDMs (Figure 6D). This demonstrates that *L. pneumophila* kills macrophages independent of the activity of apoptotic caspases, despite inhibition of host protein synthesis and the loss of mitochondrial membrane potential. Given that *L. pneumophila* can trigger pyroptosis, which can consequently activate apoptotic caspases, we finally tested whether pyroptosis is induced in late stage *L. pneumophila* infections. Δ*flaA L. pneumophila* caused delayed (~10 h) death in caspase-1/11 double deficient BMDMs compared to WT BMDMs (Figure 6E). We observed the same delayed death response in caspase-11 deficient BMDMs, suggesting that in the absence of flagellin, *L. pneumophila* triggers caspase-11 mediated pyroptosis in late stage infections (Figure 6E). Caspase-1 and -11 were not required for efficient replication (Figure 6F) or macrophage killing at higher infection rates (SI Figure 3), suggesting that besides pyroptosis *L. pneumophila* can utilize other mechanisms to induce macrophage death during egress.

## Discussion

The role of programmed host cell death signaling in *Legionella* infections has been studied extensively over the past decade. This has mainly been in the context of the caspase-1-dependent inflammatory cell death, termed pyroptosis, during invasion of macrophages. We have now utilized both host cell and bacterial genetic approaches combined with live cell imaging to more accurately define the role of apoptotic cell death in *Legionella* infections. We show that loss of the critical intrinsic apoptotic proteins, BAK and BAX, or the mPT pore component, cyclophilin D, does not significantly alter *L. pneumophila* replication or the killing of macrophages. Moreover, we also demonstrate that the genetic deletion of BCL-RAMBO or BNIP3, reported host cell pro-apoptotic molecules inhibited by the bacterial effector SidF, have no impact on *in vivo Legionella* replication. Therefore, although several *Legionella* effectors may target mitochondria and activate apoptotic caspase activity (Zhu et al., 2013), our data suggest that key mitochondrial cell death signaling pathways do not facilitate bacterial replication or retard bacterial egress, and therefore do not significantly alter *Legionella* infectivity in mice.

Several T4SS effectors have been identified that trigger sustained NF-κB signaling and consequent transcriptional up-regulation of BCL-2 family members, that includes pro-survival BCL-2 and A1 (Losick and Isberg, 2006a; Abu-Zant et al., 2007). In agreement with this, infections with virulent *L. pneumophila* renders macrophages resistant to apoptosis inducing agents (Abu-Zant et al., 2005). However, protein levels of the major pro-survival factors in infected macrophages remain stable, or are reduced, consistent with the notion that *L. pneumophila* prevents translation of most host proteins (Speir et al., 2016). Thus, *L. pneumophila* may rely on other mechanisms to sustain macrophage viability during infection. In part, this may depend on effectors that directly inhibit pro-death factor which can be activated by post-translational processes. So far, however, only one effector, SidF, has been reported to directly block the pro-apoptotic activities of BCL-RAMBO and BNIP3 (Banga et al., 2007). Genetic deletion of SidF was reported to more than double the number of apoptotic BMDMs in late stage *L. pneumophila* infections (Banga et al., 2007). To define the role of SidF and apoptosis in *L. pneumophila*-infected BMDMs, we used live-cell imaging to follow the entire infection cycle and to measure macrophage health in real time by determining plasma membrane rupture and loss of mitochondrial membrane potential. Using this technique, we did not detect significantly increased apoptosis in BMDMs infected with an *L. pneumophila* strain lacking SidF and, furthermore, show that SidF is dispensable in lung infections in mice.

BMDMs derived from C57BL/6 mice readily detect flagellin present in wild-type *L. pneumophila* and induce caspase-1-mediated pyroptotic cell death. To specifically investigate the role of SidF and apoptosis in *L. pneumophila*-infected mice, we generated a Δ*flaA*/Δ*sidF* mutant, which evades caspase-1 detection. Because the Δ*sidF* strain in the original report was on a flagellated *L. pneumophila* background, it is possible that, in the absence of SidF, flagellin reached the cytosolic sensors of NAIP5 and NLRC4 to activate caspase-1 even in permissive macrophages, albeit at reduced rates (Zamboni et al., 2006; Lamkanfi et al., 2007). The establishment and integrity of the *Legionella* containing vacuole may directly dependent on SidF and its phosphoinositide phosphatase activity which thus likely promotes efficient bacterial growth (Hsu et al., 2012). Besides triggering pyroptosis, caspase-1 may also cleave apoptotic caspases, such as caspase-3 and 7 (Amer, 2010). Our study now shows that in the absence of flagellin-mediated pyroptosis, the loss of SidF does not significantly affect the ability of *L. pneumophila* to evade apoptosis. Of note, this and the previous study used genetically different *Legionella* strains, which can affect the degree of apoptotic death as not all of the effectors are conserved and as some strains infect more efficiently (Gomez-Valero et al., 2011). It is also possible that the *L. pneumophila* strain used in this study contains additional effectors that can compensate for the loss of SidF to inhibit macrophage cell death. Nevertheless, we demonstrate that genetic deletion of the proposed SidF pro-apoptotic host cell target proteins, BCL-RAMBO, or BNIP3, also does not alter *L. pneumophila* replication, infection or host cell death kinetics. While the co-deletion of BCL-RAMBO and BNIP3 together may be required to reveal a role for these potential pro-apoptotic factors in modulating intracellular bacterial infections, the limited effects of pan-caspase inhibition or genetic loss of cyclophilin D or BAX and BAK, argue that abrogating mitochondrial death signaling does not significantly influence bacterial replication and infectivity.

*L. pneumophila* is able to directly manipulate host cell apoptotic signaling during infection. For example, *L. pneumophila*-infected cells have been reported to contain high levels of active caspase-3, but only induce host cell death with apoptotic features, such as chromosome condensation and nucleic acid fragmentation, in late stages of infection (Gao and Abu Kwaik, 1999; Abu-Zant et al., 2005). This led to the notion that *L. pneumophila* may selectively trigger apoptosis to facilitate egress (Molmeret and Abu Kwaik, 2002). At least five effectors have been identified that are able to induce apoptosis when expressed in immortalized cell lines (Zhu et al., 2013). However, their exact roles during infections remain elusive, as the co-deletion of these five effectors in *L. pneumophila* does not influence bacterial infection and intracellular replication in macrophages (Zhu et al., 2013). This is consistent with our findings showing that deletion of the essential intrinsic apoptotic executioners, BAX and BAK, does not overtly reduce the ability of *L. pneumophila* to kill macrophages or to abrogated bacterial growth (Nogueira et al., 2009). Similarly, loss of BAX/BAK and caspase-3 did not affect *L. pneumophila* growth *in vitro* (Nogueira et al., 2009). In contrast to macrophages, the above effectors are able to induce apoptosis in dendritic cells and, thus, prevent bacterial survival (Zhu et al., 2013). Bacterial growth in dendritic cells can also be restored by overexpressing pro-survival BCL-2, or by loss of BAX/BAK, suggesting that at least some effectors may act upstream of BAX/BAK (Nogueira et al., 2009). This also highlights that host cell death signaling following *L. pneumophila* infection is likely to be cell-type specific, and depend on the host cell expression levels of different cell death components.

In the absence of apoptosis, it is possible that *L. pneumophila* may induce other forms of programmed cell death to facilitate bacterial egress. While the expression of flagellin is up-regulated in late stage infections (Molmeret et al., 2010), flagellin-deficient *Legionella* species (e.g., Δ*flaA L. pneumophila* or WT *L. longbeachae*, which is naturally deficient in flagellin; Cazalet et al., 2010), are still able to efficiently kill macrophages in the late stages of infection, suggesting that this is not mediated via flagellin/caspase-1-dependent pyroptosis. Furthermore, deletion of extrinsic apoptosis (Caspase-8 deficient BMDMs) and necroptosis (RIPK3 and MLKL deficient BMDMs) did not abrogate killing of macrophages by virulent *L. pneumophila* (Speir et al., 2016). *L. pneumophila* also activates a caspase-11-dependent form of pyroptotic cell death, particularly in LPS-primed cells, to induce caspase-11 expression (Case et al., 2013; Casson et al., 2013). Cytosolic caspase-11 recognizes and binds LPS directly to either induce NLRP3/caspase-1 dependent or caspase-1 independent pyroptosis (Hagar et al., 2013; Kayagaki et al., 2013). Although caspase-11 is not required for NLRC4-dependent pyroptosis, nor for the restriction of flagellated *Legionella* infection (Cerqueira et al., 2015), there is some evidence that *Legionella* complete their terminal rounds of proliferation within the cytosol (Molmeret et al., 2007) and, thus, may activate caspase-11 upon escaping its vacuole, in order to facilitate egress. Other vacuolar pathogens also induce caspase-11-mediated death to effectively escape, as is the case in *Salmonella* Typhimurium. Caspase-11 activation is detrimental to the host as it expedites bacterial egress, allowing *S*. Typhimurium to replicate extracellularly in the absence of a caspase-1-mediated immune response (Broz et al., 2012). However, the delay in cell death after *Legionella* infections in the absence of caspase- 11 is only detectable at low infection levels. Higher numbers of bacteria must be able to trigger escape independently of, and more quickly than, caspase-11 activation alone. Furthermore, even at a low infection rates, there is no corresponding defect in bacterial replication, indicating that this delay in cell death does not limit bacterial replication. It is formally possible that multiple programmed cell death pathways are activated during *L. pneumophila* infection to facilitate bacterial egress. Alternatively, bacterial-induced killing may include other mechanisms such as the expression of lytic enzymes or overwhelming bacterial burden that lead to host cell rupture (Molmeret et al., 2002).

## Author contributions

MS and AV designed and performed experiments and interpreted data; SH, QH, GD, SM, RF generated knock out mice and revised the manuscript; JV and TN conceived the work and analyzed the data. MS, JV, and TN wrote the manuscript. All authors approved the final version of the manuscript.

## Funding

This project was funded by the National Health and Medical Research Council (Canberra, Australia) as part of the Project Grant #1024839 (JV and TN) and CDF1 Fellowship #1052598 (JV).

### Conflict of interest statement

The authors declare that the research was conducted in the absence of any commercial or financial relationships that could be construed as a potential conflict of interest.

## Supplementary material

SI Figure 1 **Genetic deletion of BCL-RAMBO. (A)** Gene targeting strategy. The targeting construct replaces exon 2 (black bar on the WT locus) with a neomycin resistance cassette (neo) resulting also in a frame-shift. Restriction sites are indicated (H, Hind III; X, XbaI; S, SacI; B, BamHI). A 3′ probe was designed to recognize a 5.9 kb and 8.7 kb fragment from Sac1-digested wild-type and mutated genomic DNA alleles, respectively. **(B)** Southern blot analysis of Sac I digested mouse tail DNA using the 3′ probe, showing the wild-type (lower, 5.9 kb) and targeted (upper; 8.7 kb) alleles of *Bcl-rambo* gene. **(C)** Western blot analysis of embryonic fibroblast cell lysates using anti-Bcl-rambo antibodies (Abcam) and anti-Actin as internal control.

SI Figure 2 ***L. pneumophila*** **replicates in BCL-RAMBO deficient macrophages**. WT and BCL-RAMBO deficient immortalized macrophages were infected with Δ*flaA* and Δ*flaA*/Δ*sidF* (MOI 10) for 2 h and the colony forming units (CFUs) determined at 6 and 48 h post infection. Mean and SD (from three independent colonies) are shown.

SI Figure 3 **Cell death of caspase-11 deficient BMDMs at high MOl**. Draq7 positive (dead) WT, caspase-1/11 DKO and caspase-11 KO BMDMs infected at a MOl of 20 with ΔflaA L. pneumophila. Data are representative of two independent experiments. Mean and S.D. of three independent biological replicates shown.

SI Video 1 **Detection of caspase-3/7 activity in cycloheximide treated macrophages**. The mitochondria of BMDMs were labeled with TMRM (red), treated with cycloheximide and incubated with Draq7 (blue DNA stain) and caspase-3/7 fluorescent substrate (green). Fluorescent and bright field images were acquired every 60 min.

SI Video 2 **Detection of caspase-3/7 activity in Δ*flaA L. pneumophila* infected macrophages**. The mitochondria of BMDMs were labeled with TMRM (red), infected with Δ*flaA L. pneumophila* and incubated with Draq7 (blue DNA stain) and caspase-3/7 fluorescent substrate (green). Fluorescent and bright field images were acquired every 60 min.

SI Video 3 **Detection of caspase-3/7 activity in WT *L. pneumophila* infected macrophages**. The mitochondria of BMDMs were labeled with TMRM (red), infected with WT *L. pneumophila* and incubated with Draq7 (blue DNA stain) and caspase-3/7 fluorescent substrate (green). Fluorescent and bright field images were acquired every 60 min.
