# Supplementary material for: Legionella pneumophila Strain 130b Evades Macrophage Cell Death Independent of the Effector SidF in the Absence of Flagellin
Source: Front Cell Infect Microbiol. 2017 Feb 16;7:35. doi: 10.3389/fcimb.2017.00035 (PMC5311068; doi:10.3389/fcimb.2017.00035)
Supplement: SI Figure 2 — L. pneumophila replicates in BCL-RAMBO deficient macrophages. WT and BCL-RAMBO deficient immortalized macrophages were infected with ΔflaA and ΔflaA/ΔsidF (MOI 10) for 2 h and the colony forming units (CFUs) determined at 6 and 48 h post infection. Mean and SD (from three independent colonies) are shown. [file Image2.PDF]

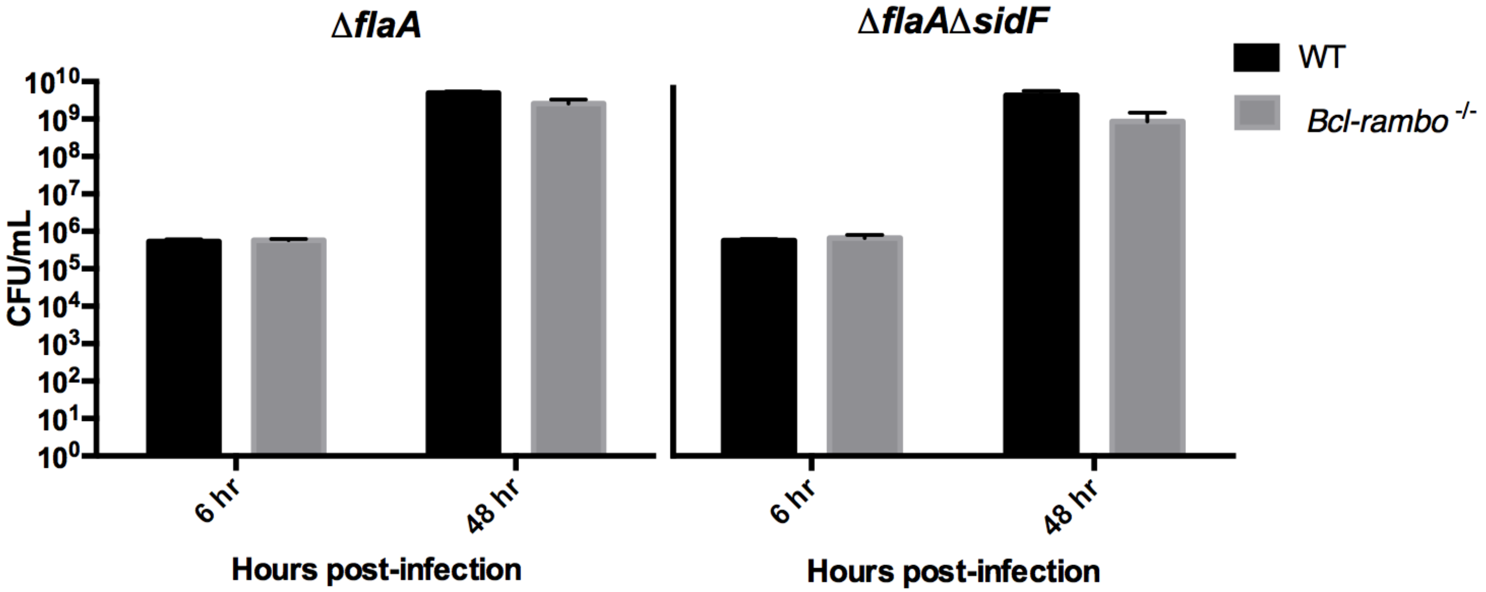

SIFig 2. ***L. pneumophila*** **replicates in Bcl-rambo deficient macrophages.** WT and Bcl-rambo deficient immortalized macrophages were infected with Δ*flaA* and Δ*flaA*/Δ*sidF* (MOI 10) for two hours and the colony forming units (CFUs) determined at 6 and 48 hours post infection. Mean and SD (from three independent colonies) are shown.
