# Supplementary material for: Legionella pneumophila Strain 130b Evades Macrophage Cell Death Independent of the Effector SidF in the Absence of Flagellin
Source: Front Cell Infect Microbiol. 2017 Feb 16;7:35. doi: 10.3389/fcimb.2017.00035 (PMC5311068; doi:10.3389/fcimb.2017.00035)
Supplement: SI Figure 3 — Cell death of caspase-11 deficient BMDMs at high MOl. Draq7 positive (dead) WT, caspase-1/11 DKO and caspase-11 KO BMDMs infected at a MOl of 20 with ΔflaA L. pneumophila. Data are representative of two independent experiments. Mean and S.D. of three independent biological replicates shown. [file Image3.PDF]

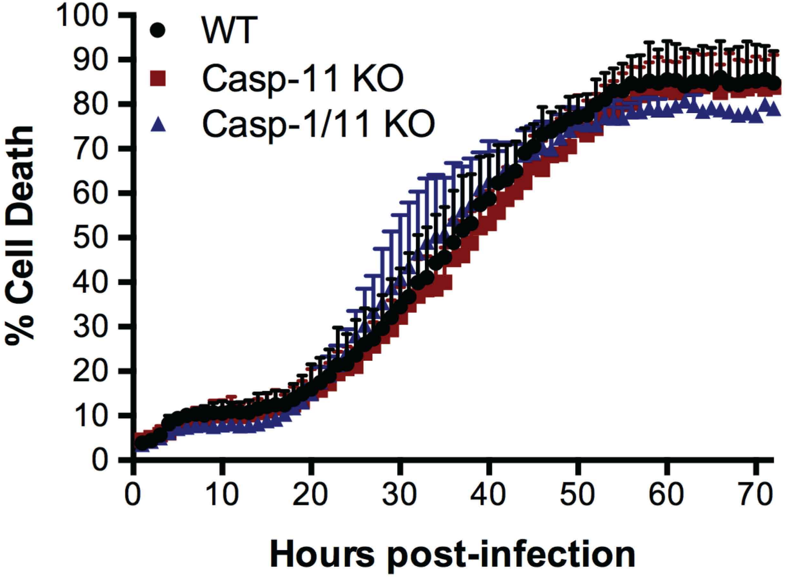

SIFig 3. Cell death of caspase-11 deficient BMDMs at high MOI.
Draq7 positive (dead) WT, Caspase-1/11 DKO and Caspase-11 KO BMDMs infected at a MOI of 20 with Δ*flaA L. pneumophila*. Data are representative of two independent experiments. Mean and S.D. of three independent biological replicates shown.
