# Supplementary material for: Legionella pneumophila Strain 130b Evades Macrophage Cell Death Independent of the Effector SidF in the Absence of Flagellin
Source: Front Cell Infect Microbiol. 2017 Feb 16;7:35. doi: 10.3389/fcimb.2017.00035 (PMC5311068; doi:10.3389/fcimb.2017.00035)
Supplement: SI Figure 1 — Genetic deletion of BCL-RAMBO. (A) Gene targeting strategy. The targeting construct replaces exon 2 (black bar on the WT locus) with a neomycin resistance cassette (neo) resulting also in a frame-shift. Restriction sites are indicated (H, Hind III; X, XbaI; S, SacI; B, BamHI). A 3′ probe was designed to recognize a 5.9 kb and 8.7 kb fragment from Sac1-digested wild-type and mutated genomic DNA alleles, respectively. (B) Southern blot analysis of Sac I digested mouse tail DNA using the 3′ probe, showing the wild-type (lower, 5.9 kb) and targeted (upper; 8.7 kb) alleles of Bcl-rambo gene. (C) Western blot analysis of embryonic fibroblast cell lysates using anti-Bcl-rambo antibodies (Abcam) and anti-Actin as internal control. [file Image1.PDF]

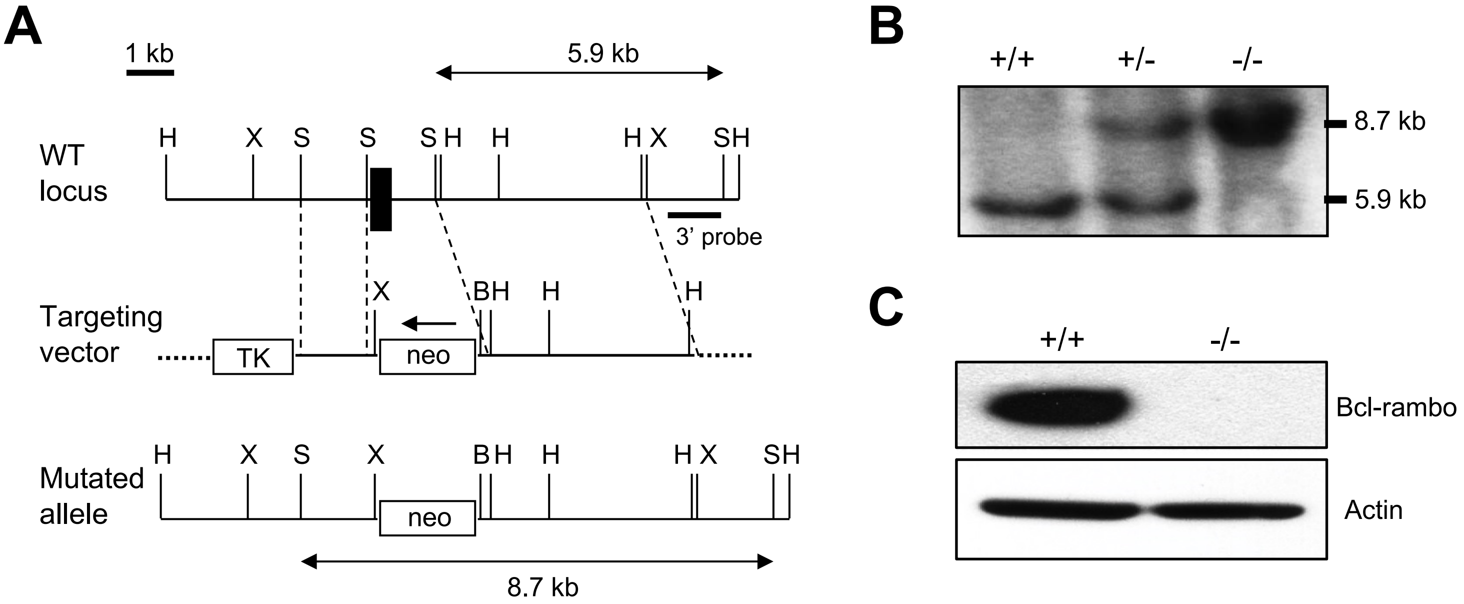

SIFig 1. Genetic deletion of BCL-RAMBO. (A) Gene targeting strategy. The targeting construct replaces exon 2 (black bar on the WT locus) with a neomycin resistance cassette (neo) resulting also in a frame-shift. Restriction sites are indicated (H: Hind III; X: XbaI; S: SacI; B: BamHI). A 3' probe was designed to recognize a 5.9 kb and 8.7 kb fragment from Sac1-digested wild-type and mutated genomic DNA alleles, respectively. **(B)** Southern blot analysis of Sac I digested mouse tail DNA using the 3' probe, showing the wild-type (lower, 5.9 kb) and targeted (upper; 8.7 kb) alleles of *Bcl-rambo* gene. **(C)** Western blot analysis of embryonic fibroblast cell lysates using anti-Bcl-rambo antibodies (Abcam) and anti-Actin as internal control.
